# A new WFD—compliant littoral macroinvertebrate index for monitoring and assessment of Mediterranean lakes (HeLLBI)

**DOI:** 10.1007/s10661-021-09493-1

**Published:** 2021-10-23

**Authors:** Efpraxia Mavromati, Dimitra Kemitzoglou, Vasiliki Tsiaoussi, Maria Lazaridou

**Affiliations:** 1 The Goulandris Natural History Museum, Greek Biotope/Wetland Centre, 14th km Thessaloniki-Mihaniona, 57001 Thermi, Greece; 2 Department of Zoology, School of Biology, Aristotle University of Thessaloniki, 54124 Thessaloniki, Greece

**Keywords:** Benthic macroinvertebrate index, Greek lakes, Eutrophication, Morphological alterations, Littoral zone, WFD

## Abstract

A new multimetric index (HeLLBI) based on littoral benthic macroinvertebrates is presented in this paper for classification of Greek natural lakes, in compliance with the requirements of Water Framework Directive (WFD). The method was developed based on the collection of littoral benthic invertebrate fauna and environmental data from 109 sampling sites in 21 natural lakes of the Greek National Water Monitoring Network. We focused the analysis on the effects of shore morphological alterations and eutrophication to the littoral invertebrate fauna, identified to family level, except oligochaetes, which were identified as a class, and more particularly to taxonomic composition and abundance, to taxa sensitivity, and to richness/diversity. Three metrics were included in the multimeric index: the relative abundance of Odonata classes, the Average Score per Taxon, and the Simpson’s diversity index. The metrics were converted to ecological quality ratios and ecological class boundaries were defined. The final multimetric index HeLLBI is expressed as an arithmetic average of normalized ecological quality ratios of the above metrics and a final score was assigned to each lake. Pressure-response relationships of HeLLBI scores were statistically tested for morphological alterations, expressed as percentage of artificial shoreline, and eutrophication, expressed as total phosphorus. The HeLLBI scores correspond to ecological classes, according to WFD, and sampling sites with different ecological status contained distinct biological communities; those at high status where more diverse and with sensitive taxa and as the water quality deteriorated, macroinvertebrate assemblages consisted of fewer and more tolerant to degradation taxa. The HeLLBI method gave a reliable assessment of littoral benthic invertebrate fauna of Greek natural lakes and could be a useful tool for the classification of ecological status of other Mediterranean lakes.

**Supplementary Information:**

The online version contains supplementary material available at 10.1007/s10661-021-09493-1.

## Introduction

Freshwater ecosystems host remarkable biodiversity; although they cover less than 1% of Earth’s surface, they host approximately one-third of vertebrate species and 10% of all species (Strayer & Dudgeon, 2010). Responding to the need for protection of surface waters in Europe, in the context of river basin management planning, the Water Framework Directive (WFD) fostered the development of ecological assessment methods for different groups of organisms (i.e., phytoplankton, macrophytes and phytobenthos, macroinvertebrates, and fish) for aquatic ecosystems (lakes, rivers, transitional waters, and coastal waters) (European Commission, 2000). The assessment methods respond to the pressures exerted on these ecosystems and aim to classify ecological status at a five-level classification scheme, indicating the degree of deviation from reference conditions (European Commission, 2000). Since the adoption of WFD, more than 300 biological assessment methods have been developed in Europe (Birk et al., 2012a), and 109 methods for lakes have been intercalibrated and included in the Member States monitoring toolkits (Poikane et al., 2019). European waters, however, still remain under pressure from a range of human activities; according to the European Environment Agency latest report (EEA, 2018), the main significant pressures on surface water bodies are hydromorphological pressures (40%), diffuse source pollution (38%), mainly coming from agriculture, and atmospheric deposition (38%), particularly related to mercury, followed by point sources (18%) and water abstraction (7%). According to the same report, the main impacts on surface water bodies are nutrient enrichment, chemical pollution, and altered habitats due to morphological changes, e.g., shoreline alterations.

Lake ecological assessments based on biological quality elements (BQEs) have mainly focused on eutrophication and acidification pressures (EEA, 2018; Poikane et al., 2019). Taking into account that in European countries, hydromorphological pressures are the second most commonly occurring types of pressures on aquatic ecosystems (after eutrophication), the need to develop methods for lakeshore assessment is important (Poikane et al., 2019). In Greece, catchment areas have undergone substantial agricultural, industrial, and urban transformation over the recent years (Kagalou, 2010), including hydromorphological alterations such as constructed outlets, roads, and embankments (Alexakis et al., 2013). Among natural lakes, intensive agriculture within their catchments seems to be a considerable land use (Mavromati et al., 2018).

Benthic macroinvertebrates have been recognized as one of the most difficult BQEs to use in ecological assessment of lakes, and so their use has been limited so far (Poikane et al., 2016). Their use, though, in national lake assessment methods from European central and alpine ecoregions has become lately more popular in evaluating not only eutrophication but also morphological degradation (Poikane et al., 2016; Urbanič et al., 2012). To our knowledge, only two national methods from Europe (Germany and Slovenia) focus mainly on hydromorphological degradation and studied their effects on benthic macroinvertebrates (Miler et al., 2013; Poikane et al., 2016; Urbanič et al., 2012). The other methods have been developed to assess hydromorphological degradation in combination with eutrophication (Gabriels et al., 2010; Miler et al., 2013; Šidagytė et al., 2013; Timm & Möls, 2012). In the Mediterranean region, existing national methods assess eutrophication, organic enrichment, and pollutants (Boggero et al., 2017; Boix et al., 2005; Spain Lakes Benthic Fauna IBCAEL, 2019). Several studies have started to include the effects of lakeshore alterations in their methodologies, most of them in Italy (Mastrantuono et al., 2008; Pilotto et al., 2015). Greece has a national method for zoobenthos from the profundal/sublittoral zone addressing eutrophication (Ntislidou et al., 2018) but lacks a zoobenthos method to reflect shoreline alterations.

The development of an efficient, widely, and easily applied assessment system based on littoral zoobenthos, with a robust and meaningful ecological interpretation, continues to be a challenge. The aim of the current research is to overcome this gap and to present a new method named as Hellenic Lake Littoral Benthic Macroinvertebrate assessment method (HeLLBI) for classification of Mediterranean natural lakes. More specifically, we describe the methodological steps to develop the method, we prove its ability to respond to morphological and eutrophication pressures, and we discuss the effectiveness of HeLLBI to assess the ecological status of Greek lakes on the basis of their littoral benthic invertebrate fauna.

## Materials and methods

### Study area and sampling procedure

**Fig. 1:**
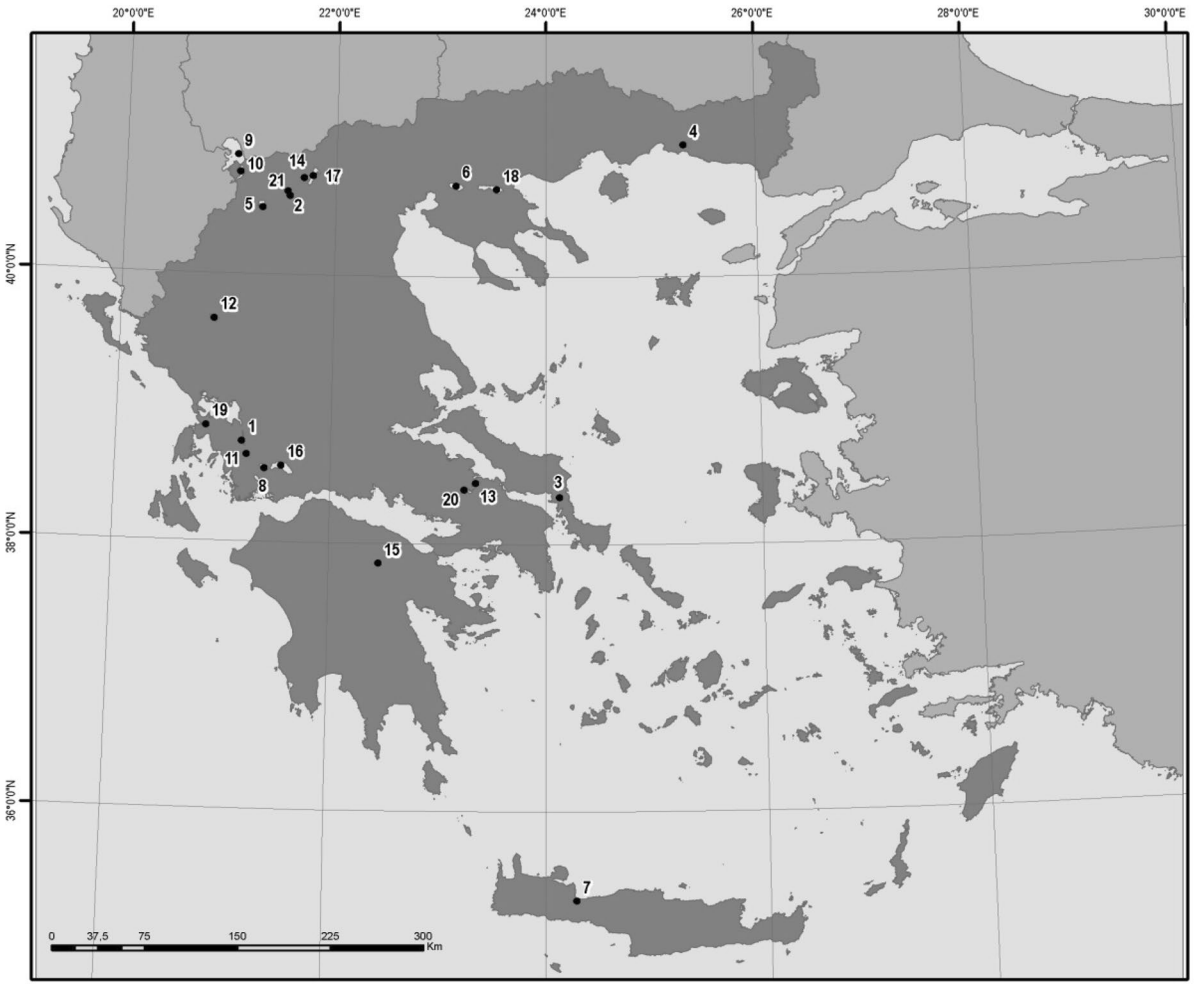
The 21 studied lakes of the Greek National Monitoring Network. 1: Amvrakia, 2: Cheimaditida, 3: Dystos, 4: Ismarida, 5: Kastoria, 6: Koroneia, 7: Kourna, 8: Lysimacheia, 9: Megali Prespa, 10: Mikri Prespa, 11: Ozeros, 12: Pamvotida, 13: Paralimni, 14: Petron, 15: Stymfalia, 16: Trichonida, 17: Vegoritida, 18: Volvi, 19: Voulkaria, 20: Yliki, and 21: Zazari

The Greek National Water Monitoring Network operates for WFD purposes and currently comprises 50 lakes with an area of 0.5 km^2^, including 24 natural ones. These natural lakes are grouped into 3 types (apart from an inland saline lake), according to the mixing regime and depth gradient (Kagalou et al., 2021). Our study was conducted in 21 lakes (Fig. 1) that belong in these 3 types, 2 of which are transboundary (Mikri Prespa and Megali Prespa).

In total, 109 littoral sampling sites have been surveyed for benthic invertebrates in 21 natural lakes in the spring season during the 2015–2018 sampling campaign. In particular, samplings took place in 2015, 2017, and 2018 (Table 1). One lake, Paralimni, was sampled three times, six lakes were sampled twice, and the rest of the lakes were sampled once. The number and location of the sampling sites for each lake were selected according to lake size, habitat maps, and land use data of the lakes and their catchment areas.

**Table 1:** Years of sampling at each lake

**Year of sampling**	**Lake**
2015	Ozeros, Kourna, Amvrakia, Yliki, Lysimacheia, Paralimni, Zazari
2017	Ozeros,Paralimni, Volvi, Petron, Megali Prespa, Kastoria, Cheimaditida, Mikri Prespa, Koroneia, Voulkaria, Dystos, Stymfalia, Trichonida
2018	Kourna, Lysimacheia Paralimni, Koroneia, Dystos, Stymfalia, Ismarida, Vegoritida, Pamvotida

In order to develop the method, the sampling strategy was designed to cover all potential types of anthropogenic modifications; to achieve that, samples were taken from unmodified sites, sites with “soft” anthropogenic modifications (relocation of rocks in the littoral zone, recreational beaches etc.), and sites with “hard” modifications (shoreline covered with concrete walls, part of urban or industrial areas) (Pilotto et al., 2015; Urbanič et al., 2012). A list of dominant substrates for the studied sites is presented in Supplement 1, Online Resource. Samplings were carried out using the 3-min kick/sweep method with standard hand net (500 μm mesh size), at the littoral zone of the lake (up to 1.2 m depth of water). The sampling effort covered proportionally all accessible mesohabitats within a stretch of 10–20 m, at each sampling site. This semi-quantitative approach is similar to methods that address the effects of lakeshore alterations with focus on the littoral benthic invertebrate community (Böhmer et al., 2014).

Samples were sieved on site and the materials were preserved in plastic bottles in 70% ethanol solution. Sorting, identification, and counting were carried out in the lab and the samples were preserved in vials containing 70% ethanol. Macroinvertebrates were hand-sorted and taxa were identified to family level, except oligochaetes which were identified as a class. The level of taxonomic identification of taxonomic groups used in this work was chosen in order to minimize the duration and the cost of sample processing. After identification, the total number of individuals of each group was recorded and the relative abundance of each group was calculated.

### Environmental parameters and pressures

Environmental data indicating eutrophication pressure were collected during the operation of the National Water Monitoring Program. Sampling campaigns were seasonal and water samples were taken at the deepest part of the lakes, from the euphotic zone by using a Nansen type sampler (De Hoyos et al., 2014). Total phosphorus (TP) was determined with persulfate digestion (APHA, 2012) and mean annual values were used in the statistical analysis for each lake. In cases where TP mean annual values were below the limit of quantification (LOQ) of the method, the value of LOQ was used in analyses. In order to assess morphological alteration, the percentage of artificial shoreline was used. Lake shorelines were initially generated by the Corine Landuse Landcover (CLC) thematic layer (version 18, classes 4.1.1 and 5.1.2; CLC, 2018). We used the Corine Land Cover (CLC) dataset for the year 2018, which is freely available from https://land.copernicus.eu/pan-european/corine-land-cover.

Particular landcover/landuse types of special interest, such as human modifications and alterations, agricultural surfaces, and natural areas, were discriminated and delineated through user photointerpretation and digitization using the Google hybrid basemap. The QGIS software was employed for the specific task. Hard engineering constructions along the shoreline (including cemented walls for bank protection) were considered as artificial shoreline and the proportion of artificial shoreline was calculated for each lake.

A number of enviromental parameters were tested as potential pressures (Supplement 2, Online Resource). The ones that were highy correlated with each other (Spearman’s rank correlation coefficient, *r*_s_ > 0.6) were excluded from the analysis. Finally, ten environmental parameters were selected: artificial shoreline (ArtShor), natural shoreline (NatShor), the percentage coverage of each lake’s catchment area by artificial land use (ARTcatch), the percentage coverage of each lake’s catchment area by intensive agriculture (IAcatch), the percentage coverage of each lake’s catchment area by low intensity agricultural areas (LIAcatch), the percentage coverage of a 50 m buffer zone by artificial land use (ART50), the percentage coverage of a 50 m buffer zone by intensive agriculture (IA50), the percentage coverage of a 50 m buffer zone by low intensity agricultural areas (LIA50), the percentage coverage of a 50 m buffer zone by natural and semi-natural areas (NSN50), and TP in water as a proxy of eutrophication. Finally, for the development of the HeLLBI method, data from all lake types were pooled together in the dataset as the samplings took place in the littoral zone, which is not used as a type descriptor. In particular, the dataset of 21 studied natural lakes is divided into three types, which are discerned only by mean depth and mixing regime: deep warm monomictic, shallow polymictic, and very shallow (Kagalou et al., 2021; Zervas et al., 2018).

### Development of the HeLLBI assessment method

#### *Selection of metrics*

According to WFD normative definitions laid out in Annex V, for lake benthic invertebrate fauna, national assessment systems should reflect taxonomic composition and abundance, ratio of disturbance sensitive taxa to insensitive taxa, and the level of diversity. More than 20 metrics, assigned to the above categories, i.e., taxonomic composition and abundance, sensitivity, and richness/diversity, were examined for possible inclusion in the assessment method (Supplement 3, Online Resource). The taxonomic composition and abundance were represented by various metrics such as the relative abundance of Odonata (% abundance classes) and combinations of Coleoptera, Ephemeroptera, Odonata, Plecoptera, and Trichoptera orders, whereas the presence of sensitive taxa was represented by the Biological Monitoring Working Party (BMWP; Armitage et al., 1983) and the Average Score Per Taxon (ASPT; Armitage et al., 1983). The Shannon–Wiener (Shannon & Weaver, 1949), Simpson (Simpson, 1949), and Margalef (Margalef, 1958) indices, as well as the number of families, were considered as richness/diversity metrics. We used metrics calculated with the relative abundance of individuals or the relative abundance classes. Seven abundance classes were discerned (AQEM CONSORTIUM, 2002) according to German Fauna Index (Böhmer et al., 2014). Boxplots for each metric were produced to check the dispersion of the dataset, extreme and zero values, and signs of skewness.

#### *Establishment of reference conditions*

Reference conditions are defined as those expected in natural or near natural state, with no or minimal disturbance and with human pressure resulting in minor effects on biological elements (European Commission, 2003). The approach to apply reference criteria was tried: results showed that although, in terms of morphology, there are un-impacted stretches of lake ecosystems with relatively low TP content, true anthropogenically un-impacted lake ecosystems, for both morphological alteration and eutrophication pressures, in Greece hardly exist (Ntislidou et al., 2018; Petriki et al., 2017). Due to the scarcity of resulting reference lakes and the lack of historical data, the reference values were derived from the national dataset (number of samples = 109) at 95th percentile of the distribution of the values of each metric. A similar approach was reported to be followed from Slovakian method for benthic invertebrates of very large rivers (Birk et al., 2012b). The resulting values were checked for suitability as reference conditions with other methods (Böhmer et al., 2014).

#### *Ecological status class boundary setting procedure*

The upper and lower anchors were set at 95th and 1th percentile respectively of all metric values in the dataset. The quality classes were calculated with equidistant division (Hering et al., 2006). The raw values of each metric were converted to EQRs by dividing the metric value with the reference value (Eq. 1).

1 $$EQRi=\frac{LAKEi}{REF}$$

2 $$\mathrm{If }EQRi\ge 1 :nEQRi=1$$

$$\begin{aligned}1&\ge EQRi\ge {EQR}_{H/G} :nEQRi\\&=\frac{\left(EQRi-{EQR}_{H/G}\right)}{\left(1-{EQR}_{H/G}\right)}x0.2+0.8\end{aligned}$$

$$\begin{aligned}{EQR}_{H/G}&\ge EQRi\ge {EQR}_{G/M} :nEQRi\\&=\frac{\left(EQRi-{EQR}_{G/M}\right)}{\left({EQR}_{H/G}-{EQR}_{G/M}\right)}x0.2+0.6\end{aligned}$$

$$\begin{aligned}{EQR}_{G/M}&\ge EQRi\ge {EQR}_{M/P} :nEQRi\\&=\frac{\left(EQRi-{EQR}_{M/P}\right)}{\left({EQR}_{G/M}-{EQR}_{M/P}\right)}x0.2+0.4\end{aligned}$$

$$\begin{aligned}{EQR}_{M/P}&\ge EQRi\ge {EQR}_{P/B} :nEQRi\\&=\frac{\left(EQRi-{EQR}_{P/B}\right)}{\left({EQR}_{M/P}-{EQR}_{P/B}\right)}x0.2+0.2\end{aligned}$$

$${EQR}_{P/B}\ge EQRi\ge 0 :nEQRi=\frac{EQRi}{{EQR}_{P/B}}x0.2$$

*EQR*_*i*_: ecological quality ratio value for each metric, as calculated for a lake *i*;

*EQR*_*H/G* or *G/M* etc._: EQR values for the corresponding boundaries, as calculated during boundary setting;

*nEQR*_*i*_: normalized EQR value for the corresponding EQR value of each metric of lake *i*.

Each metric’s EQR was converted to a normalized scale with equal class widths and standardized class boundaries, where the H/G, G/M, M/P, and P/B boundaries are 0.8, 0.6, 0.4, and 0.2, respectively (Eq. 2). This normalization was based on a linear interpolation between each class’s upper and lower boundaries (Hazewinkel, 2000). The combination of the three metrics was achieved by using the principle of equal weight for taxonomic composition and abundance, sensitivity, and richness/diversity metrics.

The final value of the HeLLBI assessment method was calculated as an arithmetic average of the normalized EQRs of the three metrics (Supplement 4, Online Resource) according to Eq. 3:

3 $$HeLLBI=\frac{\mathrm{nEQR}\text{Metric1 }+\mathrm{nEQR}\text{Metric2 }+\mathrm{nEQR}{\text{Metric3}}}{3}$$

*HeLLBI*_i_: final value of HeLLBI assessment method, which is a normalized EQR for the assessment of lake i;

nEQR_Metric1_: normalized EQR value of Metric1 for lake i.

nEQR_Metric2_: normalized EQR value of Metric2 for lake i;

nEQR_Metric3_: normalized EQR value of Metric3 for lake i.

The final score of HeLLBI can be assigned to an ecological status class according to Table 2.

**Table 2:**
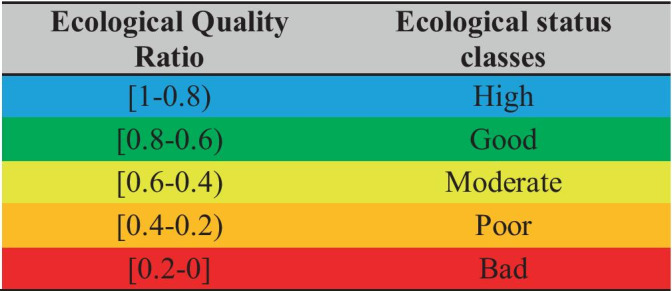
Final boundary values of HeLLBI assessment method

### Statistical assessment of method’s performance

Prior to further statistical analyses, in which temporal data were cumulated and statistically compared, the non-parametric Kruskal–Wallis test was applied to examine for differences between sampling periods. In order to evaluate the performance of the HeLLBI assessment method, the pressure-response relationships were investigated. The relationships between the final multimetric index and artificial shoreline and TP were tested by applying non-parametric Spearman’s rank correlations using IBM SPSS Statistics v.23 (IBM, 2017) software. Responses to different pressures were kept separately in the analysis.

### Description of biological communities

The similarity percentages analysis (SIMPER) was performed in order to tabulate taxa contributions to the average similarity of sampling sites within each ecological status class and to the average dissimilarity between different ecological status classes (Clarke & Gorley, 2015). The above statistical analyses were performed using Primer v7 software (Clarke & Gorley, 2015). We checked for differences in the invertebrate community composition among the five ecological classes. The indicator value index (IndVal; De Cáceres & Legendre, 2009) was applied in the dataset to investigate which families were statistically indicative of each ecological class by a permutation test. Also we used the indicators function to assess the strength and statistical significance of the relationship between taxa occurrence and groups of sites (De Caceres & Legendre, 2009). All analyses were performed with the use of ladsv (Dufrene & Legendre, 1997), indicspecies (De Cáceres & Legendre, 2009), and tidyverse (Wickam, 2017) R packages in R environoment version 3.5.3 (R Core Team, 2018).

Moreover, the ratio of sensitive to tolerant taxa was calculated for each sampling site; taxa scores are defined for Greek rivers by Lazaridou et al. (2018) for the calculation of Hellenic Evaluation Score (HES score). Therein, taxa are grouped to three categories based on their sensitivity, i.e., sensitive, medium tolerant, and tolerant and attributed a score; sensitive taxa have the highest scores, followed by medium tolerant ones and by tolerant taxa. Scores are further refined according to the relative abundance of taxa, at intervals of 0–1% (present), 1–10% (common), and > 10% (abundant). Scores are increasing with respect to the relative abundance of sensitive taxa or decreasing with respect to the relevant abundance of tolerant taxa (Supplement 5, Online Resource).

Finally, the sum of taxa scores for the studied lakes (sensitive, medium tolerant, tolerant taxa) was calculated. Taxa scores were plotted against the calculated HeLLBI values to quantify the responses of different taxonomic groups of benthic macroinvertebrates along the pressure gradient.

## Results

### Benthic macroinvertebrate fauna

Our dataset included 127,184 invertebrates from 79 taxa (including Oligochaete class). The most abundant taxa were Corixidae (36,080 individuals [ind]) and Gammaridae (33,487 ind). The taxonomic groups with higher numbers of taxa were Diptera (14), Gastropoda (10), and Coleoptera (10).

### Anthropogenic pressures

The values of TP concentrations and the percentages of artificial shoreline calculated for each lake are presented in Table 3. The TP values varied among lakes, ranging from the lowest value of TP (< 10 μg/l) in Lake Kourna to 753 μg/l in Lake Zazari. In 9 out of 21 lakes, no types of artificial shoreline were recorded. Two urban lakes (Lakes Kastoria and Pamvotida) had the highest percentage (45.7 and 30.7, respectively), whereas constructions of a lakeside village resulted in a high percentage in Lake Zazari (10.7%). A recently constructed dyke in Lake Koroneia has resulted in much of its overall artificial shoreline (15.6%).

**Table 3:** TP (annual mean values) and artificial shoreline values estimated for the studied lakes. When the same lakes were sampled in different years, the average of TP annual mean values is given. When TP values were < LOQ, this is noted. In Lakes Kourna and Paralimni, values are recorded in separate lines, due to the existence of different LOQs values

**Lake**	**TP (μg/l)**	**Artificial shoreline (%)**
Amvrakia	12	0.0
Cheimaditida	56	2.3
Dystos (2017 & 2018)	53	0.0
Ismarida	246	0.0
Kastoria	34	45.7
Koroneia (2017 & 2018)	204	15.6
Kourna (2015)	< 10	0.0
Kourna (2018)	< 29	
Lysimacheia (2015 & 2018)	57	0.0
Megali Prespa	< 29	0.0
Mikri Prespa	< 29	3.0
Ozeros (2015 & 2017)	30	0.0
Pamvotida	80	30.7
Paralimni (2015)	14	0.0
Paralimni (2017)	< 29	
Paralimni (2018)	< 29	
Petron	53	0.2
Stymfalia (2017 & 2018)	< 29	0.0
Trichonida	< 29	2.5
Vegoritida	40	0.1
Volvi	67	0.7
Voulkaria	88	0.8
Yliki	16	0.4
Zazari	753	10.7

### Generation of final multimetric index

Three metrics were included in the multimetric index development: (a) the relative abundance of Odonata classes, (b) the ASPT, and (c) the Simpson’s diversity index. The original boundary values of each metric are presented in Supplement 6, Online Resource.

**Fig. 2:**
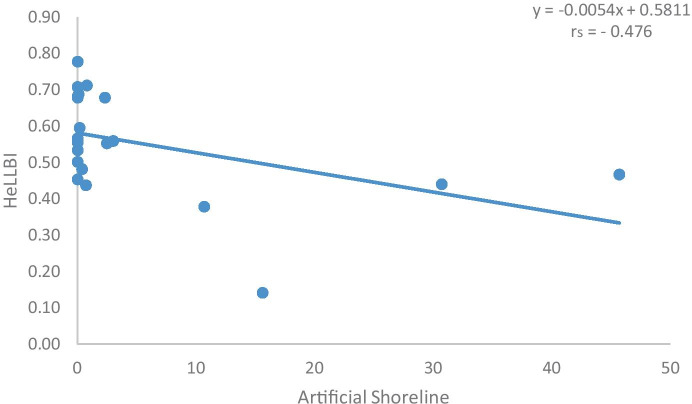
Pressure-response curve of HeLLBI in relation to artificial shoreline (percentage)

**Fig. 3:**
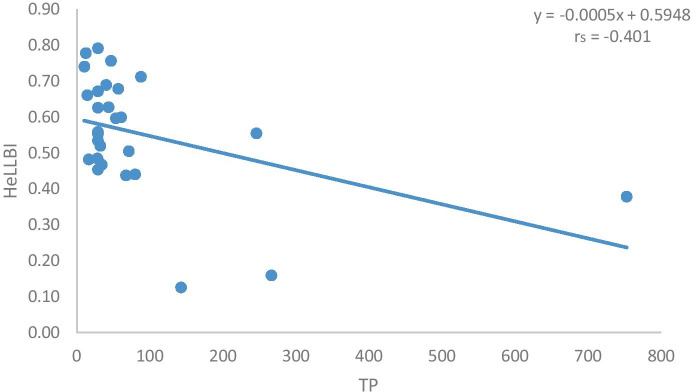
Pressure-response curve of HeLLBI in relation to TP (μgr/l)

The non-parametric Kruskal–Wallis test indicated no significant differences between sampling periods (*p* = 0.136). In order to evaluate the performance of the HeLLBI assessment method in addressing morphological alteration and eutrophication pressures, the pressure-response relationships were examined. The two pressures selected were the percentage of lake artificial shoreline and TP and were plotted against the final values of the HeLLBI method (Figs. 2 and 3). Both artificial shoreline and TP showed statistically significant correlations with final HeLLBI values via Spearman rank correlations. In particular, the correlation of HeLLBI with artificial shoreline was *r*_s_ =  − 0.476 and the correlation of HeLLBI with TP was *r*_s_ =  − 0.401.

### Biological communities

The description of biological communities of benthic invertebrates in different ecological status classes was possible using the dataset from 109 sampling sites. The results of the SIMPER analysis are given in Tables 4, 5, and Supplement 7 (Online Resource).

**Table 4:** SIMPER results for average similarity between sites within each ecological status class (Primer 7 software)

**Ecological status**	**Similarity (%)**
High	35.9
Good	44.9
Moderate	39.3
Poor	40.1
Bad	69.8

**Table 5:** SIMPER results for average dissimilarity between different ecological status classes (Primer 7 software)

**Groups of different ecological status**	**Average dissimilarity (%)**
High & bad	77.4
Good & bad	77.2
Moderate & bad	72.7
High & poor	67.0
High & moderate	64.8
Poor & bad	64.4
Good & poor	63.6
Good & moderate	61.1
High & good	60.7
Moderate & poor	60.2

Benthic macroinvertebrate communities at high status exhibited high diversity of taxa, including sensitive ones (e.g., Libellulidae from Odonata). The SIMPER analysis showed that Chironomidae, Oligochaeta, Gammaridae, and Coenagrionidae taxa contributed the most to the average similarity of sites classified with a high status. Benthic macroinvertebrate communities at good status slightly differed from undisturbed communities in composition and abundance. The numbers of families belonging to the Ephemeroptera and Odonata orders were still high, but the proportion of sensitive families to tolerant ones was lower. Libellulidae were still present but in lower abundances. According to SIMPER, Chironomidae, Corixidae, and Caenidae contributed most to the average similarity of sites classified with a good status. In the remaining three ecological status classes, Chironomidae contributed most to the average similarity of the sites; in sites classified with a bad status, they represented 97.1% of all taxa.

Indicator species analysis showed that four taxa were indicators of sites at high status, two were indicators of sites at good status, and one was an indicator of sites at bad sites (Table 6). The indicators function revealed the best combinations of indicator taxa for each ecological group (Table 7).

**Table 6:** Indicator taxa for each ecological group. The significance of the indicator value is shown: * = *p* ≤ 0.05, **p ≤ 0.01

**Indicator taxa**		
**High status**	**Good status**	**Bad status**
Libellulidae**	Caenidae*	Chironomidae**
Coenagrionidae**	Lymnaeidae*	
Gomphidae**		
Palaemonidae**

**Table 7:** Selected taxa combinations of indicator taxa for each ecological status. Prediction power, sensitivity, and indicator values (square rooted) of each taxa combination

**Status class**	**Selected taxa combination**	**Predictive power**	**Sensitivity**	**Square root of indicator value**
High status	Libellulidae	0.84	0.79	0.81
Good status	Caenidae + Coenagrionidae	0.63	0.82	0.82
Moderate status	Gammaridae + Oligochaeta	0.53	0.66	0.59
Poor status	Ceratopogonidae	0.53	0.32	0.41
Bad status	Ceratopogonidae + Chironomidae + Psychodidae	0.80	0.14	0.33

The ratios of sensitive to tolerant taxa at all 109 sampling sites are presented in Table 8. As expected, the highest number was observed in sampling sites with high status. As the water quality deteriorated, the ratio of sensitive to tolerant taxa decreased, down to bad status, where only tolerant taxa were present and the value of the ratio was zero.

**Table 8:**
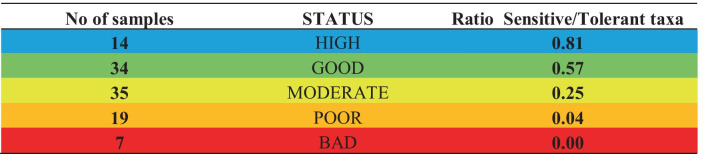
Ratio of sensitive to tolerant taxa per ecological status class

**Fig. 4:**
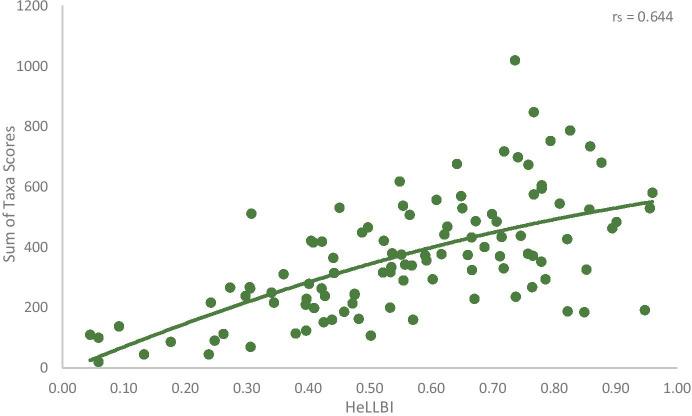
Scatterplot between sum of taxa scores and HeLLBI EQR values at 109 sampling sites. The line represents polynomial adjustment

In Fig. 4, the sum of taxa scores for the studied lakes, plotted against HeLLBI values, is shown. A clear pattern of increasing taxa scores with increasing HeLLBI values was observed.

## Discussion

The WFD requires the development of river basin management plans that should outline the set of measures required to achieve good ecological status for all water bodies. In this respect, the target of ecological assessment methods should be the detection of anthropogenically induced changes in biological communities which deviate from reference conditions (European Comission, 2000; Free et al., 2009).

Our results show that HeLLBI is a fully WFD-compliant assessment method that classifies Greek lakes according to their ecological status, based on the effects of shoreline alteration and eutrophication on littoral macroinvertebrates. According to EU WFD normative definitions (European Commission, 2000), assessment methods based on macroinvertebrate assemblages should reflect taxonomic composition and abundance, sensitivity, and diversity/richness of benthic invertebrate fauna.

In order to describe the taxonomic composition of the macroinvertebrate community, most methods use the relative abundance and taxa numbers of certain zoological groups, i.e., Ephemeroptera, Trichoptera, Plecoptera, and Odonata (Böhmer et al., 2014; Miler et al., 2013). In the development of our index, the taxonomic composition was expressed by the metric % Odonata abundance classes; we used abundance classes to avoid the influence of dominant taxa with dense populations (Miler et al., 2013). The presence of sensitive taxa was expressed by the metric ASPT, a widely used metric based on taxon-specific scores, sensitive to environmental and anthropogenic changes (Gabriels et al., 2010). Šidagytė et al. (2013) pointed out that ASPT is not affected by the sampling effort and seasonal changes and is considered as the most common sensitivity scoring system. Estonia and Lithuania also included ASPT in their methods (Ozoliņš et al., 2021; Šidagytė et al., 2013), whereas the relationships between ASPT and eutrophication were reported in the revised Danish macroinvertebrate index for lakes (Poikane et al., 2016; Wiberg-Larsen & Rasmussen, 2020).

Diversity metrics are widely used in multimetric indices as the reduction of taxa richness is highly associated with numerous environmental stressors occuring in water bodies (Gabriels et al., 2010). Τhe most frequent metrics of richness/diversity are Shannon–Wiener diversity index, Margalef diversity index, and number of families/taxa (Böhmer et al., 2014; Gabriels et al., 2010; Miler et al., 2013; Timm & Möls, 2012; Urbanič et al., 2012). Ntislidou et al. (2018) also used Simpson’s diversity index as richness/diversity metric in the development of the multimetric index GLBiI for the assessment of the ecological quality of Greek lakes, based on profundal and sublittoral benthic macroinvertebrates. Most bioassessment methods in Europe consist of metrics based on sensitivity/tolerance and richness/diversity; few methods use metrics of taxonomic composition and function, whereas abundance metrics are not included in any multimetric index, except for relative abundance (Poikane et al., 2016).

The concept of reference conditions describes the benchmark against which current conditions are compared when assessing the ecological status of water bodies (Poikane et al., 2010). There are a number of approaches to set reference conditions, such as multimetric, multivariate, landscape-context statistical modelling, and paleolimnology (Soranno et al., 2011). The most common is the reference sites approach (Poikane et al., 2010), whereas other approaches have also been used in the context of WFD, such as hindcasting modelling (Argillier et al., 2013; Ntislidou et al., 2018; Petriki et al., 2017) and calculation of percentiles (Birk et al., 2012b). In this study, in order to determine reference values of the newly developed multimetric index, we calculated the 95th percentile of the metrics in the dataset. This decision was based on the limited number of natural lakes in Greece (Mavromati et al., 2018) and the resulting absence of an adequate number of reference sites for both morphological and eutrophication pressures (Petriki et al., 2017), and the lack of historical data. With regard to the structure of the dataset of littoral zoobenthos, these 21 studied natural lakes are divided into three types, which are discerned only by mean depth and mixing regime: deep warm monomictic, shallow polymictic, and very shallow (Kagalou et al., 2021; Zervas et al., 2018). Šidagytė et al. (2013) also concluded that reference values were not affected by lake types differentiated by mean depth.

Pressure-response relationships are crucial as they provide linkages between ecological status and pressures, which can be used for management measures (Poikane et al., 2019). Most European assessment methods address eutrophication (97 of 109); only recently, few countries started to include metrics which address hydromorphlogical pressures (Poikane et al., 2019). Greece is one of them as a fish-based index was developed for Greek lakes based on its response to eutrophication and morphological alterations (Petriki et al., 2017).

The HeLLBI assessment method showed statistically significant relationships with artificial shoreline and TP, as proxies for morphological alterations and eutrophication, respectively. The Spearman rank correlations were above 0.4 in both cases. Benthic macroinvertebrates are the most commonly used group of organisms when it comes to assess hydromorphological alterations and especially the ones from the littoral zone, followed by fish and macrophytes (Poikane et al., 2019). Moreover, as Donohue et al. (2009) also indicated, the relative ease of sample collection and, therefore, the ability of cost-effective monitoring programs resulted in the majority of national methods using littoral, rather than profundal, invertebrates. In the littoral zones, habitat heterogeneity results in higher species diversity compared to sublittoral and profundal zones (White & Irvine, 2003). The decrease of taxon richness is highly associated with the deterioration of habitat diversity of the littoral zone, which could further affect the whole lake ecosystem (Brauns et al., 2007; McGoff et al., 2013). Therefore, the community composition of littoral macroinvertebrates is a powerful tool to assess anthropogenic pressures (Pilotto et al., 2015). Indeed, many studies have reported shifts in the taxonomic composition of benthic invertebrates in response to shore alterations (Böhmer et al., 2014; Miler et al., 2013; Urbanič, 2014; Urbanič et al., 2012). In our analysis, sites at high status are characterized by families of Odonata, like Libellulidae. Libellulidae showed dinstict preference at high status and they were identified as indicator for this ecological group. Families of Odonata, which are considered to be habitat specialists, are thought to be the most affected by human shoreline development (Brauns et al., 2007). Miler et al. (2013) argued that Odonata taxa are considered to be more appropriate in lake’s assessment methods, in comparison with other biological groups like Plecoptera, as they are severely affected by littoral habitat deterioration. They found high negative correlation with their stressor index and they included Odonata in metric selection of LIMCO and LIMHA (Miler et al., 2013). Coenagrionidae from Odonata are associated with natural shorelines, as high relative abundance of this family in natural littoral habitats with submerged macrophytes has been recorded in other studies (Brauns et al., 2007; Pilotto et al., 2015). In our study, Coenagrionidae along with Caenidae are characterized as indicator taxa of sites at good status. On the other end of the spectrum, Chironomidae characterize mostly sites at bad, poor, and moderate status. According to our results, Oligochaeta and Chironomidae showed high contribution to the SIMPER similarity and characterized several ecological classes, as they include several species with a taxon-specific response to eutrophication (Dorić et al., 2020; Verdonschot, 2006). In particular, at sites with bad ecological status, Chironomidae were the dominant family and they were characterized as indicator taxa of these sites. Chironomidae consist of taxa with different ecological preferences; in polluted environments, they usually dominate the benthic community (Lazaridou et al., 2018). Moreover, higher abundances of Chironomidae along an increase in proportion of artificial shoreline were observed in our results. Brauns et al. (2007) also found increasing abundances of Chironomidae with increasing proportion of artificial shoreline. Therefore, macroinvertebrates might be the most appropriate biological group to evaluate habitat loss associated with shoreline alteration (Free et al., 2009). However, Brauns et al. (2007) argue that sites with retaining walls and beaches, due to their soft human modification, do not exhibit clear community structure associated with these types of shorelines. According to SIMPER results, the percentage of dissimilarity was higher than 60% between all different groups of ecological status which showed that each status was associated with a dinstict macroinvertebrate community, as also reported by Lazaridou et al. (2018). These communities mainly consist of tolerant, medium tolerant, and sensitive taxa to habitat degradation. The scores assigned to these taxa are based on their sensitivity to different kinds of pollution such as point source or diffuse pollution in rivers (Artemiadou & Lazaridou, 2005). Although originally designed and applied in rivers, our results indicate that they can be useful in lakes too as higher HeLLBI values were associated with increasing taxa scores. In particular, the ratio of sensitive to tolerant taxa showed a clear decrease from high towards bad ecological status. At bad status, the value of the ratio was zero as only tolerant taxa were present. Except Chironomidae, only two other taxa contributed to the similarity of sites at bad status: Ceratopogonidae and Oligochaeta, which generally are well known as tolerant organisms. According to Pilotto et al. (2015), Ceratopogonidae are associated with eutrophic conditions and Oligochaeta are representative of fine sediments and strongly associated with softly altered sites. Artemiadou and Lazaridou (2005) also characterized Ceratopogonidae as medium tolerant taxa for Greek rivers.

The IndVal (De Cáceres & Legendre, 2009) showed no indicator taxa for sites at moderate and poor status classes. This analysis enables the identification of indicator taxa for a specific group of sites (Pilotto et al., 2015). De Cáceres et al. (2012) suggested that a group of sites with no indicator taxa is probably because of its unique macroinvertebrate assemblage in relation to the other groups of sites. The extended version of indicator values analysis (indicspecies; De Cáceres & Legendre, 2009) deals with this particularity and provides a set of indicators (either a single taxon or taxa combinations) that show high predictive value for the target site group. The highest predictive values were found at sites at high and bad status, 0.84 and 0.80, respectively. Sites at high status highlighted Libellulidae as a single indicator taxon with also high sensitivity, which is an estimation of the frequency of the family at these sites. Palaemonidae were also selected as indicator at high status; they were only described in Lake Kourna (Christodoulou et al., 2016), a lake treated as a reference in other biological quality elements (Zervas et al., 2018). A combination of taxa with high sensitivity and rather high predictive power were Caenidae and Coenagrionidae which were found at sites at good status. Caenidae were strongly associated with sites at good status, as both SIMPER and IndVal analyses reported the same results. Pilotto et al. (2015) found correlation of some species of Caenidae with stones and altered shorelines. Indicspecies analysis also showed that the combination of Gammaridae and Oligochaeta indicates sites at moderate status, although Gammaridae are characterized as sensitive according to SIMPER analysis and other studies in the Mediterranean region (Ntislidou et al., 2018; Pilotto et al., 2015). Sites at poor status were represented by Ceratopogonidae with relatively low sensitivity, as this taxon is also part of the taxa combination of sites at bad status. The last taxa combination, represented by Ceratopogonidae, Chironomidae, and Psychodidae, can predict sites at bad status but with extremely low sensitivity which is attributed to the fact that these families can be found also in other status classes. In our study, Ceratopogonidae were found in both poor and bad status which showed their preference to eutrophic conditions. Some taxa may be related to more than one group but when a combination is reported in a status class, it means that it can predict its target group with accuracy (De Cáceres et al., 2010).

The development of a cost-effective procedure in routine monitoring programs has been a challenge to ecologists for many years and the level of taxonomic resolution used for macroinvertebrate assemblages is often debatable in terms of accuracy and bioassessments outcomes (Birk et al., 2012a; Jones, 2008; Urbanič, 2014). Birk et al. (2012a) pointed out that the degree of taxonomic resolution used in a study is highly associated with the purpose of the study, the available expertise, and logistics to implement the study. The type of the sampling and the high level of taxonomic identification of zoological groups used in this work were chosen to minimize the processing time and the cost in the context of WFD national monitoring. On the other hand, there is a certain loss of information if the identification is to family level, because some taxonomic groups contain genera or species, which exhibit a wide range of environmental tolerance, and, consequently, they respond differently to human disturbances. The identification below the family level (genera or species) or a tiered taxonomic approach, in which taxa known to be sensitive are identified primarily at genus or species and the remaining taxa only to family, would be more efficient (in terms of accuracy) for biomonitoring purposes. For instance, it is known that genera and species within Chironomidae exhibit a wide range of environmental tolerance and their taxonomic identification to family tends to prevent their correct use as bioindicators (Dorić et al., 2020; Ntislidou et al., 2018). Bazzanti et al. (2012) pointed out the need to include more chironomid-dependent metrics in lake bioassessment studies based on their high correlation with eutrophication despite the difficulties presented in genus or species identification. According also to Dorić et al. (2020), Chironomidae should be included in lake trophic classification systems as long as their abundances and their lower taxonomic levels are included in the analysis. The same remark can be also made for Oligochaeta identified as class: three families (Naididae, Tubificidae, and Lumbriculidae) are generally dominant in the littoral of lakes and each of them indicates different ecological conditions (Lazaridou et al., 2018; Ntislidou et al., 2018). Studies showed that when lower taxonomic resolution is used, the results are more robust and less biased, especially in the case of Oligochaeta (Verdonschot, 2006). Our study focused on the selection of the most appropriate indicators with high discriminatory power and low error, in order to monitor the stressors affecting ecosystem conditions. The balance between cost-effective yet efficient assessment method is always a struggle but the use of higher taxonomic resolution seemed to be a valid compromise to achieve that. Urbanič (2014) tested for differences in macroinvertebrate community response to lakeshore modification between the lowest available level and family level and his results showed that there were not any major differences.

In recent years, the awareness of the ecological functions of intact littoral zones has grown in practical lake management (Porst et al., 2015). A well validated multimetric index which provides results at a whole lake level will help environmental managers to propose measures to further improve the ecological status of water bodies when needed. The application of HeLLBI in the context of WFD national monitoring will help to better understand the relationships between morphological alterations and biological communities. Moreover, it can be further developed and/or modified by including other suitable metrics and by testing its performance for other hydromorphological pressures. Some adjustments and refinements of the index can be also considered in order to separate the macroinvertebrate community responses to eutrophication from those generated by morphological alteration. The disentangling of different pressures would also be useful in order to apply targeted management measures in the context of river basin management plans (Kagalou & Latinopoulos, 2020). Finally, a possible future challenge would be to study lakes using temporal samplings to assess the stability of the proposed index in time.

## Conclusions

The WFD-compliant HeLLBI assessment method based on littoral benthic invertebrate fauna in Mediterranean lakes in Greece was developed from data of the Greek National Water Monitoring Network. It consists of three metrics, the relative abundance of Odonata classes, the Average Score per Taxon, and the Simpson’s diversity index. The HeLLBI method reflects in a satisfactory manner the response of benthic invertebrate fauna of lake littoral zone to artificial shoreline and TP and assesses subsequent changes in the ecological status of lakes. The differences in the invertebrate community composition were investigated among the five ecological classes. HeLLBI gave a reliable assessment of littoral benthic invertebrate fauna of Greek lakes and could be a useful tool for the classification of ecological status of other Mediterranean lakes. In the light of new data, future improvements in this assessment method may be required.

## Supplementary Information

Below is the link to the electronic supplementary material.

Supplementary file1 (DOCX 52 KB)
