# Supplementary material for: A new WFD—compliant littoral macroinvertebrate index for monitoring and assessment of Mediterranean lakes (HeLLBI)
Source: Environ Monit Assess. 2021 Oct 23;193(11):745. doi: 10.1007/s10661-021-09493-1 (PMC8536626; doi:10.1007/s10661-021-09493-1)
Supplement: Supplementary file 1 — Supplementary file1 (DOCX 52 KB) [file 10661_2021_9493_MOESM1_ESM.docx]

Electronic Supplementary Material

A new WFD - compliant littoral macroinvertebrate index for monitoring and assessment of Mediterranean lakes (HeLLBI)

Efpraxia Mavromati^1^, Dimitra Kemitzoglou^1^, Vasiliki Tsiaoussi^1^ and Maria Lazaridou^2^

^1^The Goulandris Natural History Museum, Greek Biotope/Wetland Centre, 14th km Thessaloniki - Mihaniona, 57001 Thermi, Greece; [emavromati@ekby.gr](mailto:emavromati@ekby.gr)

^2^Department of Zoology, School of Biology, Aristotle University of Thessaloniki, 54124, Thessaloniki, Greece

Supplement 1. List of dominant substrates at 109 sampling sites. Oz: Ozeros, Kou: Kourna, Am: Amvrakia, Yl: Yliki, Lys: Lysimacheia, Par: Paralimni, Zaz: Zazari, Vol: Volvi, Pet: Petron, MegP: Megali Prespa, Kas: Kastoria, Ch: Cheimaditida, MikP: Mikri Prespa, Kor: Koroneia, Voul: Voulkaria, Dys: Dystos, St: Stymfalia, Tr: Trichonida, Is: Ismarida, Veg: Vegoritida, Pam: Pamvotida

| **Site** | **Dominant substrate** |
| --- | --- |
| Oz615 | sand |
| Oz715 | sand/cobbles |
| Oz617 | sand/gravel |
| Oz717 | sand |
| Kou615 | sand |
| Kou715 | sand |
| Kou118 | sand/cobbles |
| Kou218 | sand/gravel |
| Kou318 | sand |
| Kou418 | sand |
| Kou518 | sand |
| Kou618 | sand |
| Kou718 | sand |
| Am815 | sand |
| Am915 | sand |
| Am1015 | sand |
| Yl915 | sand |
| Yl1015 | sand |
| Lys115 | cobbles |
| Lys215 | sand |
| Lys315 | sand |
| Lys118 | sand/gravel |
| Lys218 | sand/silt |
| Lys318 | sand/silt |
| Lys418 | sand/silt |
| Lys518 | sand/silt |
| Lys618 | sand |
| Par315 | sand |
| Par415 | sand/gravel |
| Par515 | cobbles |
| Par517 | sand/gravel |
| Par617 | cobbles |
| Par717 | sand |
| Par118 | sand/silt |
| Par218 | sand/gravel |
| Par318 | sand |
| Par418 | sand |
| Par518 | silt |
| Par618 | sand/silt |
| Par718 | silt |
| Par818 | sand |
| Zaz415 | sand |
| Zaz515 | cobbles |
| Vol117 | sand/gravel |
| Vol217 | sand |
| Vol317 | boulder/gravel |
| Vol417 | sand |
| Vol517 | sand |
| Vol617 | sand |
| Pet117 | sand/silt |
| Pet217 | gravel |
| Pet317 | silt |
| MegP117 | gravel |
| MegP217 | sand |
| MegP317 | sand |
| Kas117 | sand/ silt |
| Kas217 | gravel |
| Kas317 | gravel |
| Kas417 | sand/gravel |
| Kas517 | silt |
| Ch117 | silt |
| Ch217 | silt |
| Ch317 | silt |
| Ch417 | boulder |
| MikP117 | sand/gravel |
| MikP217 | sand |
| MikP317 | gravel |
| Kor117 | silt |
| Kor217 | sand |
| Kor317 | silt |
| Kor118 | sand |
| Kor218 | sand |
| Kor318 | sand/silt |
| Kor418 | silt |
| Kor518 | silt |
| Kor618 | sand |
| Voul117 | sand |
| Voul217 | sand/gravel |
| Dys117 | sand/silt |
| Dys217 | sand |
| Dys118 | sand/silt |
| Dys218 | sand |
| Dys318 | sand |
| St117 | silt |
| St118 | sand |
| Tr117 | cobbles |
| Tr217 | sand/gravel |
| Tr317 | cobbles |
| Tr417 | sand/gravel |
| Tr517 | gravel |
| Tr617 | sand/gravel |
| Tr717 | sand/gravel |
| Tr817 | sand/gravel |
| Tr917 | silt |
| Is118 | sand |
| Veg118 | sand/silt |
| Veg218 | sand/silt |
| Veg318 | sand |
| Veg418 | sand |
| Veg518 | sand/gravel/cobble |
| Veg618 | gravel/cobble |
| Veg718 | gravel |
| Pam118 | silt |
| Pam218 | gravel/cobble |
| Pam318 | boulder |
| Pam418 | gravel/cobble |
| Pam518 | silt |
| Pam618 | boulder/cobble |
| Pam718 | gravel/cobble |

Supplement 2. List of candidate environmental and pressure parameters estimated during the present study for the index development

| **Environmental parameters** | **Abbreviation** |
| --- | --- |
| Total Phosphorus (μg/l) | TP |
| Artificial land use: a) the sum of all categories of CLC class 1 (urban areas continuous and discontinuous, industrial and commercial zones, communication infrastructures and networks, mines, etc.), % on existing CLC coverage | ARTcatch |
| Intensive agriculture: a) the sum of CLC categories corresponding to a high potential impact from agricultural activities: arable land (including irrigated land), permanent crops (with associated annual crops), vineyards, orchards, olive groves, complex cultivation patterns, CLC codes: 2.1, 2.2, 2.4.1, 2.4.2, % on existing CLC coverage | IAcatch |
| Low intensity agricultural areas: a) the sum of CLC categories corresponding to a lower potential impact from agricultural activities: pastures, land principally occupied by agriculture, with significant areas of natural vegetation, agro-forestry areas, CLC codes: 2.3.1, 2.4.3, 2.4.4, % on existing CLC coverage | LIAcatch |
| Natural and semi-natural areas: a) the sum of CLC categories corresponding to forest and natural areas, wetlands, water bodies, CLC codes: 3.1.1, 3.1.2, 3.1.3, 3.2, 3.3, 4 and 5, % on existing CLC coverage | NSNcatch |
| LUL-Catchment | LUL |
| Artificial land use: a) the sum of all categories of CLC class 1 (urban areas continuous and discontinuous, industrial and commercial zones, communication infrastructures and networks, mines, etc.), % on existing CLC coverage (50m buffer) | ART50 |
| Intensive agriculture: a) the sum of CLC categories corresponding to a high potential impact from agricultural activities: arable land (including irrigated land), permanent crops (with associated annual crops), vineyards, orchards, olive groves, complex cultivation patterns, CLC codes: 2.1, 2.2, 2.4.1, 2.4.2, % on existing CLC coverage (50m buffer) | IA50 |
| Low intensity agricultural areas: a) the sum of CLC categories corresponding to a lower potential impact from agricultural activities: pastures, land principally occupied by agriculture, with significant areas of natural vegetation, agro-forestry areas, CLC codes: 2.3.1, 2.4.3, 2.4.4, % on existing CLC coverage (50m buffer) | LIA50 |
| Natural and semi-natural areas: a) the sum of CLC categories corresponding to forest and natural areas, wetlands, water bodies, CLC codes: 3.1.1, 3.1.2, 3.1.3, 3.2, 3.3, 4 and 5, % on existing CLC coverage (50m buffer) | NSN50 |
| Artificial land use: a) the sum of all categories of CLC class 1 (urban areas continuous and discontinuous, industrial and commercial zones, communication infrastructures and networks, mines, etc.), % on existing CLC coverage (100m buffer) | ART100 |
| Intensive agriculture: a) the sum of CLC categories corresponding to a high potential impact from agricultural activities: arable land (including irrigated land), permanent crops (with associated annual crops), vineyards, orchards, olive groves, complex cultivation patterns, CLC codes: 2.1, 2.2, 2.4.1, 2.4.2, % on existing CLC coverage (100m buffer) | IA100 |
| Low intensity agricultural areas: a) the sum of CLC categories corresponding to a lower potential impact from agricultural activities: pastures, land principally occupied by agriculture, with significant areas of natural vegetation, agro-forestry areas, CLC codes: 2.3.1, 2.4.3, 2.4.4, % on existing CLC coverage (100m buffer) | LIA100 |
| Natural and semi-natural areas: a) the sum of CLC categories corresponding to forest and natural areas, wetlands, water bodies, CLC codes: 3.1.1, 3.1.2, 3.1.3, 3.2, 3.3, 4 and 5, % on existing CLC coverage (100m buffer) | NSN100 |
| Artificial Shoreline | ArtShor |
| Natural Shoreline | NatShor |

Supplement 3. List of candidate benthic metrics considered for the index development. Abundance classes (AC): AC 1=1-2 individuals, AC 2=3-10 individuals, AC 3=11-30 individuals, AC 4=31-100 individuals, AC 5=101-300 individuals, AC 6=301-1000 individuals, AC 7 > 1000 individuals

| **Metrics** | **Abbreviation** |
| --- | --- |
| Number of families | NoFam |
| ASPT | ASPT |
| BMWP (Biological Monitoring Working Party) | BMWP |
| Simpson's diversity index | Simpson |
| Shannon-Wiener diversity index | Shannon |
| Margalef diversity | Margalef |
| Relative Abundance of Oligochaeta (%) | Oligochaeta (%) |
| Relative Abundance of Chironomidae (%) | Chironomidae (%) |
| Relative Abundance of EPT (abundance classes) | EPT (%AC) |
| Relative Abundance of EPT | EPT |
| Relative Abundance of ECO (abundance classes) | ECO (%AC) |
| Relative Abundance of ECO | ECO |
| Relative Abundance of ETO (abundance classes) | ETO (%AC) |
| Relative Abundance of ETO | ETO |
| Relative Abundance of EPO (abundance classes) | EPO(%AC) |
| Relative Abundance of EPO | EPO |
| Relative Abundance of EPC | EPC |
| Relative Abundance of Odonata (abundance classes) | Odonata (%AC) |
| Relative Abundance of Odonata (%) | Odonata (%) |
| Relative Abundance of Gastropoda (%) | Gastropoda (%) |
| Relative Abundance of Mollusca (%) | Mollusca (%) |
| Relative Abundance of Bivalvia (%) | Bivalvia (%) |

Supplement 4. Overview of EQRs of the three final metrics at 109 sampling sites

| **Site** | **EQR Odonata (%AC)** | **EQR ASPT** | **EQR Simpson** |
| --- | --- | --- | --- |
| Oz715 | 0.00 | 0.68 | 0.23 |
| Oz617 | 0.00 | 0.38 | 0.76 |
| Kou118 | 0.00 | 0.63 | 0.69 |
| Kou518 | 0.00 | 0.63 | 0.19 |
| Yl915 | 0.00 | 0.53 | 0.66 |
| Lys115 | 0.00 | 0.67 | 0.99 |
| Lys315 | 0.00 | 0.31 | 0.43 |
| Lys318 | 0.00 | 0.50 | 0.72 |
| Lys418 | 0.00 | 0.42 | 0.84 |
| Vol417 | 0.00 | 0.63 | 0.80 |
| Vol517 | 0.00 | 0.63 | 0.09 |
| Vol617 | 0.00 | 0.68 | 0.69 |
| MegP217 | 0.00 | 0.63 | 0.69 |
| MegP317 | 0.00 | 0.63 | 0.56 |
| Kas117 | 0.00 | 0.53 | 0.49 |
| Kas217 | 0.00 | 0.55 | 0.72 |
| Kas417 | 0.00 | 0.67 | 0.23 |
| Ch317 | 0.00 | 0.50 | 1.00 |
| MikP217 | 0.00 | 0.57 | 0.92 |
| Kor117 | 0.00 | 0.17 | 0.00 |
| Kor217 | 0.00 | 0.17 | 0.00 |
| Kor317 | 0.00 | 0.13 | 0.65 |
| Kor118 | 0.00 | 0.17 | 0.10 |
| Kor218 | 0.00 | 0.13 | 0.00 |
| Kor318 | 0.00 | 0.17 | 0.00 |
| Kor418 | 0.00 | 0.50 | 0.21 |
| Kor518 | 0.00 | 0.25 | 0.28 |
| Kor618 | 0.00 | 0.50 | 0.53 |
| St118 | 0.00 | 0.60 | 1.00 |
| Tr217 | 0.00 | 0.70 | 0.86 |
| Tr317 | 0.00 | 0.48 | 0.79 |
| Tr417 | 0.00 | 0.45 | 0.46 |
| Tr517 | 0.00 | 0.99 | 0.79 |
| Tr617 | 0.00 | 0.59 | 0.49 |
| Tr917 | 0.00 | 0.46 | 0.98 |
| Is118 | 0.00 | 0.71 | 0.95 |
| Veg318 | 0.00 | 0.88 | 0.83 |
| Veg618 | 0.00 | 0.78 | 0.68 |
| Pam118 | 0.00 | 0.13 | 0.27 |
| Pam318 | 0.00 | 0.50 | 0.91 |
| Pam418 | 0.00 | 0.29 | 0.89 |
| Pam518 | 0.00 | 0.29 | 0.62 |
| Pam618 | 0.00 | 0.50 | 0.78 |
| Z515 | 0.14 | 0.67 | 0.11 |
| Z415 | 0.15 | 0.78 | 0.43 |
| MikP117 | 0.15 | 0.63 | 0.92 |
| Yl1015 | 0.19 | 0.74 | 0.78 |
| MikP317 | 0.19 | 0.66 | 1.00 |
| Vol317 | 0.19 | 0.63 | 0.40 |
| Tr717 | 0.19 | 0.83 | 0.86 |
| Kas517 | 0.20 | 0.66 | 0.81 |
| Veg118 | 0.21 | 0.66 | 0.79 |
| Vol117 | 0.21 | 0.84 | 0.15 |
| Vol217 | 0.24 | 0.78 | 0.91 |
| Pet117 | 0.27 | 0.76 | 0.19 |
| St117 | 0.27 | 0.69 | 0.64 |
| Lys215 | 0.32 | 1.00 | 0.83 |
| MegP117 | 0.32 | 0.81 | 0.43 |
| Voul117 | 0.33 | 0.67 | 0.81 |
| Oz717 | 0.34 | 0.85 | 0.79 |
| Am815 | 0.39 | 0.88 | 1.00 |
| Oz615 | 0.41 | 0.79 | 0.80 |
| Par415 | 0.43 | 0.63 | 0.55 |
| Dys117 | 0.44 | 0.49 | 0.40 |
| Pam218 | 0.44 | 0.60 | 0.96 |
| Kou418 | 0.46 | 0.82 | 0.73 |
| Veg218 | 0.48 | 0.85 | 0.89 |
| Kou615 | 0.55 | 0.57 | 0.49 |
| Par418 | 0.55 | 0.77 | 0.98 |
| Dys217 | 0.55 | 0.76 | 0.97 |
| Tr817 | 0.55 | 0.77 | 0.80 |
| Lys218 | 0.56 | 0.78 | 0.30 |
| Dys318 | 0.57 | 0.74 | 0.98 |
| Pam718 | 0.57 | 0.74 | 0.75 |
| Am1015 | 0.58 | 0.85 | 1.00 |
| Par617 | 0.59 | 0.74 | 0.91 |
| Lys118 | 0.60 | 0.83 | 0.95 |
| Par717 | 0.60 | 0.99 | 0.27 |
| Kou618 | 0.60 | 0.69 | 0.13 |
| Am915 | 0.62 | 0.81 | 0.87 |
| Par218 | 0.62 | 0.80 | 0.89 |
| Lys518 | 0.65 | 0.65 | 0.79 |
| Par515 | 0.67 | 0.69 | 0.41 |
| Par518 | 0.67 | 0.68 | 0.81 |
| Par718 | 0.67 | 0.70 | 0.99 |
| Kas317 | 0.67 | 0.81 | 0.67 |
| Ch217 | 0.67 | 0.86 | 0.30 |
| Ch117 | 0.68 | 0.90 | 0.75 |
| Veg718 | 0.68 | 0.80 | 0.86 |
| Par818 | 0.69 | 0.88 | 0.76 |
| Ch417 | 0.71 | 1.00 | 0.77 |
| Par318 | 0.73 | 0.98 | 1.00 |
| Par618 | 0.77 | 0.88 | 0.98 |
| Pet317 | 0.78 | 0.69 | 0.66 |
| Voul217 | 0.78 | 0.80 | 0.88 |
| Pet217 | 0.80 | 0.63 | 0.59 |
| Dys118 | 0.91 | 0.77 | 0.27 |
| Par315 | 0.93 | 0.77 | 0.88 |
| Veg518 | 0.94 | 1.00 | 0.27 |
| Par517 | 0.95 | 0.77 | 0.23 |
| Lys618 | 0.98 | 0.74 | 0.96 |
| Kou715 | 1.00 | 1.00 | 0.84 |
| Kou218 | 1.00 | 0.94 | 0.52 |
| Kou318 | 1.00 | 0.76 | 0.80 |
| Kou718 | 1.00 | 0.76 | 0.79 |
| Par118 | 1.00 | 0.70 | 0.51 |
| Dys218 | 1.00 | 0.78 | 0.79 |
| Tr117 | 1.00 | 1.00 | 0.88 |
| Veg418 | 1.00 | 1.00 | 0.87 |

Supplement 5. Taxa scores related to the increase of relative abundance for the calculation of Hellenic Evaluation Score (HES score) (modified from Artemiadou and Lazaridou, 2005)

| **Sensitivity** | **Taxa** | **Present**  **(0-1%)** | **Common**  **(1.01-10%)** | **Abundant**  **(>10%)** |
| --- | --- | --- | --- | --- |
| **Sensitive taxa** | Siphlonuridae, Phryganeidae | 100 | 110 | 120 |
|  | Perlodidae | 90 | 97 | 100 |
|  | Leptoceridae, Polycentropodidae, Limnephilidae, Ecnomidae, Aeshnidae, Lestidae, Corduliidae, Libellulidae Hydraenidae | 80 | 86 | 90 |
| **Medium**  **Tolerant**  **taxa** | Stratiomyidae, Hydrobiidae | 70 | 75 | 78 |
|  | Platycnemididae, Gomphidae, Tabanidae, Ceratopogonidae, Empididae, Elmidae Viviparidae, Neritidae | 60 | 64 | 67 |
|  | Caenidae, Hydropsychidae, Gammaridae, Corophiidae, Atyidae, Dryopidae, Helophoridae, Psychodidae | 50 | 53 | 56 |
| **Tolerant taxa** | Ephemerellidae, Baetidae, Hydroptilidae, Tipulidae, Dolichopodidae, Limoniidae, Haliplidae, Curculionidae, Chrysomelidae, Piscicolidae, Glossiphoniidae | 40 | 38 | 35 |
|  | Coenagrionidae, Chironomidae, Dytiscidae, Hydrophilidae, Corixidae, Mesoveliidae, Pleidae, Naucoridae, Notonectidae, Asellidae, Ostracoda, Physidae, Bithyniidae, Thiaridae Hirudinidae,Sphaeriidae, Oligochaeta | 30 | 25 | 20 |
|  | Muscidae, Ephydridae, Chaoboridae, Lymnaeidae, Planorbidae, Erpobdellidae | 20 | 12 | 3 |
|  | Valvatidae | 10 | 2 | 1 |

Supplement 6. Original boundary values of individual metrics of the HeLLBI assessment method

| **Metric** | **Odonata (%AC)** | **ASPT** | **Simpson** |
| --- | --- | --- | --- |
| Reference | 16.67 | 5.47 | 0.80 |
| H/G | 13.33 | 4.68 | 0.64 |
| G/M | 10.00 | 3.88 | 0.48 |
| M/P | 6.67 | 3.09 | 0.32 |
| P/B | 3.33 | 2.29 | 0.16 |

Supplement 7**.** Summary table of the SIMPER results for benthic taxa contribution to similarity between sites (PRIMER 7 Software)

| Ecological status: High, n=14  (Similarity: 35.92%) | | | Ecological status: Good, n=34 (Similarity: 44.92%) | | | Ecological status: Moderate, n=35  (Similarity: 39.33%) | | | Ecological status: Poor, n=19 (Similarity: 40.13%) | | | Ecological status: Bad, n=7 (Similarity: 69.84%) | | |
| --- | --- | --- | --- | --- | --- | --- | --- | --- | --- | --- | --- | --- | --- | --- |
| Taxa | % | %Cum. | Taxa | % | %Cum. | Taxa | % | %Cum. | Taxa | % | %Cum. | Taxa | % | %Cum. |
| Chironomidae | 26.97 | 26.97 | Chironomidae | 26.79 | 26.79 | Chironomidae | 33.50 | 33.50 | Chironomidae | 46.10 | 46.10 | Chironomidae | 97.13 | 97.13 |
| Oligochaeta | 13.25 | 40.22 | Corixidae | 16.16 | 42.95 | Oligochaeta | 17.99 | 51.49 | Oligochaeta | 16.15 | 62.26 |  |  |  |
| Gammaridae | 11.18 | 51.41 | Caenidae | 12.70 | 55.65 | Gammaridae | 16.95 | 68.43 | Corixidae | 15.75 | 78.01 |  |  |  |
| Coenagrionidae | 8.96 | 60.37 | Coenagrionidae | 9.75 | 65.40 | Corixidae | 14.73 | 83.17 | Gammaridae | 11.93 | 89.93 |  |  |  |
| Corixidae | 8.57 | 68.94 | Gammaridae | 8.17 | 73.57 | Caenidae | 6.22 | 89.39 | Caenidae | 3.41 | 93.35 |  |  |  |
| Caenidae | 8.40 | 77.34 | Oligochaeta | 7.73 | 81.29 | Asellidae | 1.69 | 91.08 | Ceratopogonidae | 2.05 | 95.40 |  |  |  |
| Libellulidae | 6.14 | 83.47 | Atyidae | 4.18 | 85.48 | Erpobdellidae | 1.54 | 92.62 |  |  |  |  |  |  |
| Atyidae | 4.88 | 88.35 | Baetidae | 3.02 | 88.49 | Hydracarina | 1.27 | 93.89 |  |  |  |  |  |  |
| Dreissenidae | 1.98 | 90.33 | Asellidae | 2.13 | 90.62 | Atyidae | 1.16 | 95.05 |  |  |  |  |  |  |
| Baetidae | 1.87 | 92.20 | Ceratopogonidae | 1.52 | 92.15 |  |  |  |  |  |  |  |  |  |
| Asellidae | 1.63 | 93.83 | Lymnaeidae | 1.38 | 93.53 |  |  |  |  |  |  |  |  |  |
| Physidae | 1.22 | 95.05 | Libellulidae | 1.09 | 94.63 |  |  |  |  |  |  |  |  |  |
|  |  |  | Dreissenidae | 0.97 | 95.60 |  |  |  |  |  |  |  |  |  |
